# FRET Visualization of High Mechanosensation of von Willebrand Factor to Hydrodynamic Force

**DOI:** 10.3390/bios15040248

**Published:** 2025-04-14

**Authors:** Mingxing Ouyang, Yao Gao, Binqian Zhou, Jia Guo, Lei Lei, Yingxiao Wang, Linhong Deng

**Affiliations:** 1 Institute of Biomedical Engineering and Health Sciences, School of Medical and Health Engineering, Changzhou University, 1 Gehu Rd, Wujin District, Changzhou 213164, China; 2 School of Pharmacy, Changzhou University, Changzhou 213164, China; 3 Shu Chien-Gene Lay Department of Bioengineering, and Institute of Engineering in Medicine, University of California at San Diego, La Jolla, CA 92093, USAywang283@usc.edu (Y.W.); 4 Alfred E. Mann Department of Biomedical Engineering, University of Southern California, Los Angeles, CA 90089, USA

**Keywords:** von Willebrand factor, fluid shear, Förster resonance energy transfer (FRET), mechanosensation, molecular unfolding, hemophilia

## Abstract

von Willebrand factor (vWF) is a large glycoprotein in the circulation system, which senses hydrodynamic force at vascular injuries and then recruits platelets in assembling clots. How vWF mechanosenses shear flow for molecular unfolding is an important topic. Here, a Förster resonance energy transfer (FRET) biosensor was developed to monitor vWF conformation change to hydrodynamic force. The vWF-based biosensor is anchored on the cell surface, in which the A2 domain is flanked with a FRET pair. With 293T cells seeded into microfluidic channels, 2.8 dyn/cm^2^ of shear force (i.e., 28 μN/cm^2^, or 264.1/s in shear rate) induced a remarkable FRET change (~60%) in 30 min. A gradient micro-shear below 2.8 dyn/cm^2^ demonstrated FRET responses positively related to flow magnitudes, with 0.14 dyn/cm^2^ (1.4 μN/cm^2^) inducing an obvious change (~16%). The FRET increases indicate closer positioning of A2’s two terminals in vWF or the addition of a more parallel orientation of the FRET pair, supported with the high FRET of the A2-only-based biosensor, which probably resulted from flow-induced A2 dissociation from vWF intramolecular binding such as that in A1/A3 domains. Interestingly, gradient flow increases from 2.8 to 28 dyn/cm^2^ led to decreasing FRET changes, suggesting the second-level unfolding in the A2 domain. The LOCK-vWF biosensor with bridged A2 two terminals or an A2-only biosensor could not sense the shear, indicating a structure-flexible A2 and large vWF molecules that are important in the mechanosensation. In conclusion, the developed vWF-based biosensor demonstrated the high mechanosensation of vWF with two-level unfolding to shear force: the dissociation of the A2 domain from vWF intramolecular binding under a micro-shear, and then the unfolding of A2 in vWF under a higher shear; the FRET response to shear force at a very low scale may support the observed clot formation at microvascular wounds. This study provides new insights into the vWF’s mechanosensitive feature for its physiological functions and implicated disorders.

## 1. Introduction

Hemophilia is a hereditary bleeding disorder caused by the deficiency or abnormal function of coagulation factors [1,2,3]. Under normal conditions, when a blood vessel is damaged, coagulation factors are activated and form a thrombus, thus preventing bleeding. However, in hemophiliacs, the synthesis or function of coagulation factors is affected due to mutations in specific coagulation factor genes [4,5]. von Willebrand disease (vWD) is a common disorder among hemophiliacs and is caused by an insufficient amount of or the abnormal function of von Willebrand factor (vWF) in plasma [6,7]. vWF is a macromolecular glycoprotein, the gene of which is located on human chromosome 12, containing 52 exons and 51 introns, and these exons encode the different structural domains of the vWF protein [8,9].

The most common types of mutations in vWD are point mutations which may result in structural abnormalities or the impaired function of the vWF protein [10]. For example, some point mutations may lead to increased cleavage of the vWF protein, thereby reducing its concentration in the blood, while others may affect the ability of the vWF protein to bind to platelets and coagulation factor VIII, thereby affecting the blood coagulation process [11,12]. Large deletion or insertion mutations may also lead to the complete or partial deletion of the vWF gene, which can cause severe vWD symptoms [13,14]. In conclusion, the development of vascular hemophilia is closely associated with mutations in the vWF gene.

vWF is a polysaccharide protein with a molecular weight of 220 kDa and it is secreted by endothelial cells and megakaryocytes. The mature vWF protein consists of 2050 amino acid residues including four major structural domains, D’-D3-A1-A2-A3-D4-C1-C2-C3-C4-C5-C6-CK, which interact with a wide range of molecules and cell types, forming multimers through head-to-tail disulfide bonding and molecular weights of up to several million Daltons [15,16,17]. This multimerization property is critical to the functionality of vWF as it increases binding to platelets and collagen [18,19]. At vascular injuries, vWF binds to exposed collagen through its A3 structural domain and promotes platelet adhesion by binding to the glycoprotein GPIb-IX complex on the platelet surface through the A1 domain; vWF also acts as a carrier for factor VIII, together ensuring that hemostasis and thrombosis occur at the appropriate time [20,21,22]. vWF’s A2 structural domain is able to bind to ADAMTS13, a metalloproteinase, leading to the cleavage of vWF, thereby regulating vWF size and activity [15,23,24].

In normal circulation, vWF exists in a globular structure and does not bind to platelets. However, when the vasculature is damaged, exposed collagen and the local high-shear environment induce a conformational change in vWF that exposes sites for binding to the platelet membrane GPIb-IX complex, forming a platelet–vWF–collagen complex. This process is particularly significant under high-shear conditions, as shear enhances the binding of vWF to collagen [25,26,27,28]. Additionally, in a high-shear environment, larger vWF multimers unfold more readily, exposing more binding sites and thus enhancing their ability to promote platelet adhesion and aggregation [29,30]. Here, our work was trying to understand how vWF mechanosenses the microfluidic shear force for molecular unfolding, which is a critical step to initiate coagulation and thrombus assembly.

The effects of fluid shear on physiological function are widely used in a variety of fields including vascular health, bone development, cell biology, and epithelial cell research. In physiology, fluid shear stress (FSS), which is a frictional force generated by blood, lymph, or other biological fluids on the walls of tubes or cell surfaces, is crucial in several physiological processes, ranging from vascular health and bone development to the biological function of epithelial cells [31,32,33]. Understanding and mastering these basic laws and mechanisms is not only important for basic science research, but also provides a theory and practical direction for the development and optimization of clinical treatment strategies. We developed a shear force biosensor based on the nature of vWF molecules in this work, which can be applied to characterize the fluidic conditions experienced by cells.

As a large multimer, vWF is able to sense changes in shear forces in blood flow and plug ruptures in the vessel. The mechanisms behind this mechanosensing process are complex to study by conventional methods. A study with single-molecule imaging and high-pressure, fast-switching microfluidics revealed electrostatic guidance in speeding flow-activated vWF binding to GPIbα and recruiting platelets under flow conditions [34]. Single-molecule force measurements obtained by atomic force microscopy (AFM) demonstrated a strong interaction within vWF dimers, which dissociates at forces above 50 pN and provides ~80 nm of dimer elongation [35]. An Aptamer-based single molecular study with optical tweezers revealed the unfolding of only the A1 domain under 3–7 pN of stretch force [36]. In type 2B von Willebrand disease, the mutations in the A1 domain resulted in vWF inappropriate binding to platelet GPIBα with elongated bond lifetimes [37]. The intramolecular A1/A2 binding has an auto-inhibitory effect on vWF recruiting platelets under normal circulation conditions, which can be unfolded by shear force [38,39].

The in vitro study showed that vWF multimerized through sequential stacking steps is assembled as a right-hand helical tubular storage, and clinical mutations of vWF in disrupting the assembly may lead to von Willebrand disease [40]. The molecular length of vWF is regulated by shear-induced A2 domain unfolding, the crystal structure of which shows a functional adaptation as a shear sensor [41]. Specifically, the usual structure of A domains in vWF contains the hydrophobic six β-strands encircled by six α-helices, whereas for the A2 domain, the α4-helix is replaced by a loose loop, with poor packing around the central β4-strand [41]. This feature in the A2 structure may narrow the range of forces at which unfolding occurs, or they may slow the rate of refolding. The structure reveals von Willebrand disease mutations that are hypothesized to reduce the force of A2 unfolding. Our designed biosensor would utilize the A2 domain in shear force sensing in spite of the large size of vWF.

It has been well recognized that the plastic vWF mechanosenses the hydrodynamic force used to induce conformation change and elongated unfolding, which exerts its coagulation function to form a complex with collagen and platelets. The previous studies visualized the elongation of vWF molecules under high shear forces, such as hundreds of dyn/cm^2^ [34,38,42]. In this work, we applied Förster (fluorescence) resonance energy transfer (FRET) technology to study the conformation change in vWF in response to shear force, in which the A2 domain is flanked with an ECFP (enhanced cyan fluorescent protein) and YPet (yellow fluorescent protein for energy transfer) pair within full-length vWF. FRET has high sensitivity in measuring the subtle spatial changes within the molecules [43,44], which provided a suitable way to visualize the mechanosensation of vWF in the physiological shear condition. ECFP and YPet have been an optimized pair with high FRET efficiency [45]. By anchoring the vWF-based FRET biosensor on the surface of the cell plasma membrane, our work revealed the two-level unfolding of vWF in response to shear force, in which a flexible A2 domain and the large-molecular size of vWF are important contributing factors. The developed vWF-based biosensor demonstrated the high mechanosensation of vWF to the shear force even at a low scale, which may support clot formation at microvascular wounds.

## 2. Materials and Methods

### 2.1. Cell Types and Sources

Human Embryonic Kidney (HEK293T) cells are epithelial cells derived from human embryonic kidneys, purchased from BeiNa Biologics. The cells grow faster with enhanced adhesiveness and are modified to contain the SV40 antigen, which enables them to express certain types of vectors and genes more efficiently, and are widely used in transfection experiments with high transfection efficiency [46]. The 293T cells were used to present the vWF-based biosensor on the cell surface in order to conduct the flow experiments in this work.

### 2.2. Main Reagents and Instruments

High-glucose DMEM medium, fetal bovine serum (FBS), Opti-MEM medium, 0.25% trypsin-EDTA, cell detachment digestion reagent Accutase, and transfection kit Lipofectamine 3000 were purchased from Themo Fisher Scientific. The human full-length vWF in the pcDNA3.1 vector was ordered from Addgene [47]. Hank’s balanced salt solution (HBSS) was obtained from Procell, Wuhan, China; the single-channel flow chamber slides were from ibidi, Germany, and the flow rate peristaltic pumps were from Shanghai Feigeweit Company. The FRET microscopy system and inverted microscope Primo Vert were purchased from Zeiss, Oberkochen, Germany.

### 2.3. Constructions of vWF-Based FRET Biosensor and Related Mutant Plasmids

In the first step, the target fragment of the A2 domain (Pro^1490^-Cys^1670^) from human full-length vWF, the N-terminal of which contains 7 amino acid residues before the β1-Strand as the flexible linker to ECFP, was amplified by PCR using pCS-CG-WT-Cer-vWF as the template [47]; the linear vector fragment of pcDNA3.1-ECFP-YPet without the Camodulin-M13 part was obtained by PCR using the pcDNA3.1-Ca^2+^-YPet biosensor as the template [43]. The two linear fragments were ligated by a Gibson Assembly kit (NEB) to obtain plasmid pcDNA3.1-ECFP-A2-YPet. The primers used in this step were designed according to the sequences of the ligated genes, and A2 domain amplification used the following PCR primers: forward primer 1 (FP1): ttc gtg acc gcc gcc CCC AAG AGG AAC TCC and reverse primer 1 (RP1): ttc acc ttt aga cat ACC GGT ACA ACA ACT. The linear pcDNA3.1-ECFP-YPet fragment amplification was conducted by using FP2: AGT TGT TGT ACC GGT atg tct aaa ggt gaa gaa tta ttc act ggt and RP2: GGA GTT CCT CTT GGG ggc ggc ggt cac gaa ctc cag (the capital letters refer to the annealing part on the template DNA).

In the second step, the ECFP-A2-YPet fragment was amplified by PCR using pcDNA3.1-ECFP-A2-YPet as the template; at the same time, pcDNA3.1-WT-vWF was used as the template for PCR to obtain the linear fragment pcDNA3.1-vWF-ΔA2 (without the A2 domain). The two fragments were ligated using the Gibson Assembly kit, and then the plasmid pcDNA3.1-vWF-ECFP-A2-YPet was obtained by replacing the A2 domain in vWF with ECFP-A2-YPet. The primers used in the second step were as follows: ECFP-A2-YPet amplification with FP3: TTG GGG GTT TCG ACC CTG GGG atg gtg agc ggc gag gag and RP3: GGT GGG GAT CTG CAG CCC CTC ttt gta caa ttc att cat acc caa ttc att cat acc ctc ggt aat acc; pcDNA3.1-vWF-ΔA2 amplification with FP4: GGT ATT ACC GAG GGT ATG AAT GAA TTG tac aaa gag ggg ctg cag atc ccc acc and RP4: CTC CTC GCC CTT GCT CAC CAT ccc cag ggt cga aac ccc caa.

In the third step, the achieved plasmid pcDNA3.1-vWF-ECFP-A2-YPet was further swapped into the vector pDisplay which can present the expressed biosensor protein on the cell membrane surface. This subcloning step was performed by a commercial company, Beijing BGI Genomics Co., to acquire the plasmid pDisplay-vWF-ECFP-A2-YPet along with sequencing confirmation. Then, the pDisplay-vWF-ECFP-LOCK-A2-YPet version was generated by the company through the mutation of Asp^1493^ and Cys^1669^ to Cys^1493^ and Gly^1669^ at the N- and C-terminals of the A2 domain, respectively. Hence, the mutated A2 structural domain forms a double-sulfide bond between its N- and C-terminals to prevent the proper confirmation change, named LOCK-A2 [47]. Specifically, the A2 sequences at N-terminal CCC AAG AGG AAC TCC and C-terminal AGT TGT TGT ACC GGT were changed to CCC AAG AGG TGC TCC and AGT GGT TGT ACC GGT. The short versions of the ECFP-A2-YPet-only and ECFP-LOCK-A2-YPet-only FRET biosensors were constructed into the pDisplay vector as derived from their full-length vWF FRET biosensors by BGI Genomics Co. (Beijing, China).

All the mentioned plasmids used in this work are ampicillin (Amp) resistant, and were amplified by the Midi plasmid extraction kit (QIAGEN, Hilden, Germany). The DNA concentrations were measured by NanoDrop (TECAN, Kawasaki, Japan), and the plasmids could be used in a series of subsequent cell transfection experiments.

### 2.4. 293T Cell Culture and Transfection with FRET Biosensors

293T cells were cultured in high-glucose DMEM (Dulbecco’s Modified Eagle Medium) medium supplemented with 10% FBS and double-antibiotics, and were placed in a 37 °C humidified incubator containing 5% CO_2_. The cells used in the experiments were generally passaged no more than 10 times. According to the transfection method of Lipofectamine 3000 liposomes (Thermo Invitrogen, Waltham, MA, USA), cells were inoculated into 24-well plates in normal culture medium without antibiotics one day before transfection, and the cell density reached 60%~80% at the time of transfection. Each well was transfected with 1 μg of plasmid DNA; after 8~12 h, the medium was replaced with fresh medium without antibiotics. Twenty-four hours after the start of transfection, cells were digested with Accutase solution, and transferred into ibidi newly unsealed or fully sterilized single-channel slides (µ-Slide I^0.4^ Luer, 0.4 mm × 5 mm × 50 mm in channel height, width and length) and replaced with fresh culture medium containing 1% FBS. The experiments of cell flow microscopy imaging were started two days after the transfection.

### 2.5. Fluid Shear Experimental Setup and FRET Microscope Imaging

Before the start of the experiment, a suitable hose (Size-14^#^) was connected to the single-channel flow chamber slide (µ-Slide I^0.4^ Luer) through a Luer fitting, and then the hose was connected to a peristaltic pump. The inlet and outlet ports were inserted into a centrifuge tube prepared with 20 mL of HBSS, and the tube was placed in a tube rack. It should be noted that before starting the fluid experiment, the HBSS in the inlet port should be pumped to the Luer connector using a peristaltic pump to ensure that the single-channel flow chamber slide is filled with the liquid and that the connector and pump are seamlessly connected to each other. This prevents air bubbles from being generated during the flow of the liquid, hence not affecting the stability of the experiment.

According to the protocol from the manufacturer ibidi, shear force calculation for µ-Slide I^0.4^ Luer is as follows: τ = η∙131.6∙Φ, of which Φ: flow rate mL/min; τ: shear stress in dyn/cm^2^; η: dynamical viscosity in dyn∙s/cm^2^. η = 0.0106 dyn∙s/cm^2^ for HBSS measured by Malvern Kinexus rheometer at room temperature.

In the FRET microscope system, the filter parameters were an excitation of (436 ± 10) nm, a dichroic mirror of 455 nm for both ECFP and FRET channels, an emission of (475 ± 20) nm and an emission of (535 ± 15) nm for the ECFP and FRET (YPet) channels, respectively. In the imaging experiments, we used an ×100 oil objective to take the FRET images. The fast switching of ECFP and FRET imaging channels was controlled by the Zeiss software (ZEN 2.3 SP1, blue edition) system to ensure the simultaneous acquisition of image data from both channels. During the experimental flow process, fluorescence images were collected at the 2 min interval, and the peristaltic pump, which had been set in advance to correspond to the experimental parameters, was turned on after 10 min of FRET imaging, and the total flow administrating time was 30 min. The images were acquired at room temperature, due to the lack of an appropriate temperature controller to fit the fluidic system in the lab yet.

### 2.6. Quantitative and Statistical Analysis of FRET Image Data

The FRET signals from the experimental image raw data were quantified by FRET image analysis software FluoCell 6.0.0 [48]. The ratios of fluorescence intensity between ECFP and FRET channels were calibrated pixel to pixel after subtracting background effects, and the ratiometric images as well as FRET/ECFP ratio data were acquired. Statistical analysis of the data was carried out in GraphPad Prism 6.0 software. Time-course curves and plots with scattering points (mean ± S.E.M.) of FRET ratios were analyzed, as well as the differences between each of the two data sets.

Differences between data sets were analyzed using a one-way ANOVA test between the control and experimental groups, and one pair analysis was performed by Student’s *t*-test. *, **, ***, and **** represent *p*-values < 0.05, 0.01, 0.001, and 0.0001 for significant differences, while “ns” indicates no significant difference.

## 3. Results

### 3.1. Design of vWF-Based FRET Biosensor and Flow Experimental Setup

vWF is a large-sized protein made by endothelial cells/megakaryocytes to be stored in Weibel–Palade bodies or stay in the circulation system [49]. vWF can sense the hydrodynamic force of the vascular vessels at an injured site, which helps unfold the protein to recruit platelets in assembling clots and stop the bleeding [39,50]. The A2 domain in vWF has been identified as one mechanosensing element during thrombus formation [47]. Studies from in vitro models visualized the elongated vWF molecules under high shear flows, such as hundreds of dynes per cm^2^ [34,38,42]. In this work, we applied FRET responses to study vWF in sensing hydrodynamic force, a research tool which has high sensitivity in monitoring the molecular conformation.

**Figure 1:**
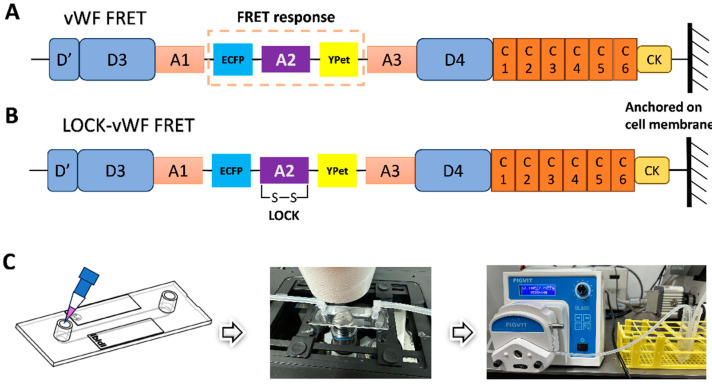
**Design of vWF-based biosensor and flow experimental setup.** (**A**,**B**) Schematic diagrams of the vWF- or LOCK-vWF-based FRET biosensor. A2 domain is flanked by ECFP and YPet, and the biosensor protein is anchored on the surface of cell plasma membrane. (**C**) The setup of flow experiments on the microscope. The image of microfluidic chamber was adapted from the manufacturer ibidi.

The FRET pair of fluorescent proteins ECFP and YPet was inserted into the full-length vWF, with the A2 domain flanked by the FRET pair (shown in Figure 1A). The vWF-based FRET biosensor was subcloned into the pDisplay vector which could direct the protein to the secretory pathway and anchor the biosensor on the surface of the cell plasma membrane by the transmembrane domain from platelet-derived growth factor receptor (PDGFR) [51]. This positioning scenario helped mimic vWF’s sensing of the hydrodynamic force while sticking to the under-endothelial layer of the vascular wall. We also prepared the mutated version by forming a disulfide bond between A2’s N- and C-terminals (LOCK-A2), named the LOCK-vWF biosensor (Figure 1B). The LOCK-A2 could resist the conformation change for vWF self-associations induced by flow shear [47].

In the fluidic and FRET microscopy setup (Figure 1C), 293T cells were seeded into the single-channel fluidic slide after the transfection of the biosensor for 24 h, and were maintained in culture medium with 1% FBS (low serum to reduce the shedding possibility of the biosensor on the cell surface). HBSS was applied for the flow, which can maintain the cell condition for hours long. The peristaltic pump was able to adjust the flow force through the chamber slide (µ-Slide I^0.4^ Luer) from 0.0027 to 178.1 mL/min within ±0.5% variation in accuracy. The FRET imaging was conducted at room temperature, and the imaging process was generally completed in 40 min.

### 3.2. Changes in vWF-Based FRET in Response to Different Shear Forces

**Figure 2:**
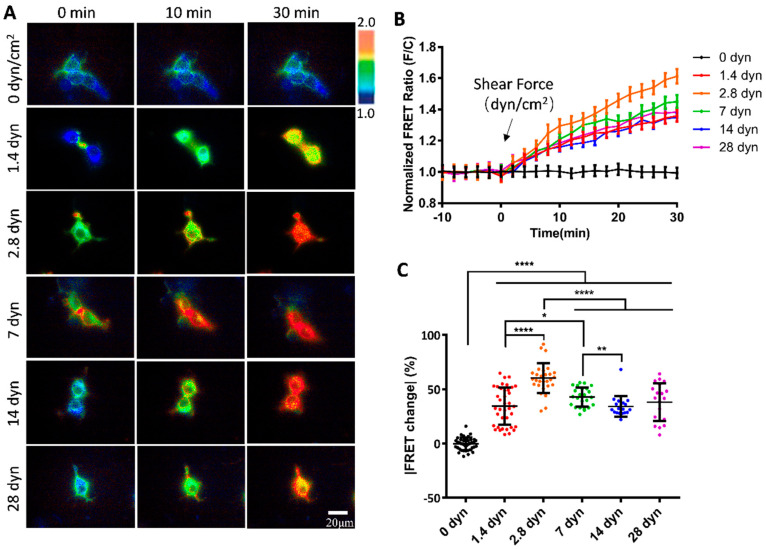
**FRET changes in vWF-based biosensor in 293T cells under gradient flow shears.** (**A**) FRET ratiometric images of cells expressing vWF-based biosensor at 0, 10 and 30 min under fluid shear of 0, 1.4, 2.8, 7, 14, and 28 dyn/cm^2^, with 0 dyn as control. (**B**) The time-course curves of FRET ratio (FRET/ECFP) quantifications (mean ± S.E.M.) for cell groups in (**A**) under indicated shear forces. (**C**) Scatter plots of quantified FRET ratio changes for vWF-based biosensor at 30 min under indicated shear forces. Experiment was repeated three times independently. Specific values (mean ± S.E.M.) in (**C**) were as follows: 0 dyn, −0.32 ± 0.90, N = 40; 1.4 dyn, 34.41 ± 2.80, N = 38; 2.8 dyn, 60.49 ± 2.55, N = 29; 7 dyn, 42.77 ± 1.81, N = 24; 14 dyn, 34.14 ± 2.20, N = 19; 28 dyn, 38.04 ± 3.89, N = 20. N refers to sample size. *, **, **** indicate *p* < 0.05, 0.01, and 0.0001 by one-way ANORA test; ‘ns’ denotes non-significant difference, and so on throughout this paper.

To examine whether the vWF-based biosensor was able to detect the hydrodynamic force, we applied different magnitudes of shear stress from 0 to 28 dyn/cm^2^ to the cells seeding in the microfluidic channels. 293T cells expressing the vWF-based biosensor on the cell surface showed apparent FRET responses to the shear flow, as presented by the FRET ratiometric images in Figure 2A (Supplementary Materials, Movie S1). Specifically, for the cells implanted on the single-channel flow chamber slides, without applying shear force to the cells, the FRET values did not change significantly (0 dyn); when the peristaltic pump was turned on to apply shear force, the biosensor showed clear FRET changes with the ratio (FRET/ECFP) increasing over time. The cells generally had more FRET changes at the cell boundaries, corresponding to the shear force exerted on the cell surface. The time-course curves from FRET/ECFP ratio quantifications on the cells demonstrated no FRET changes before flow, and gradual increases after flow applications (Figure 2B).

To compare the FRET changes under different magnitudes of shear forces, 2.8 dyn/cm^2^ (i.e., 28 μN/cm^2^) induced more changes (60%) than 1.4 dyn/cm^2^ (38%) in 30 min (Figure 2C). The FRET changes did not continue to increase under higher flow magnitudes, instead, after reaching the peak at 2.8 dyn/cm^2^, the FRET change rate started to decrease from 2.8 to 28 dyn/cm^2^ (Figure 2C). Based on the FRET efficiency calibrations, these results indicate that the two terminals of the A2 domain in vWF were becoming closer spatially under a low shear force (0–2.8 dyn/cm^2^), and then becoming more separated at a higher shear force (2.8–28 dyn/cm^2^). The observations from FRET measurements seemed to point to the unfolding of the vWF at two stages: at a low shear force, A2 dissociated from vWF intramolecular binding such as in A1/A3 domains, and then under a higher shear force, the A2 domain was unfolded for cleavage accessing by ADAMTS13. The underlying mechanism requires further investigation.

### 3.3. Sensitive FRET Responses of the vWF-Based Biosensor to Micro-Shear Flow

**Figure 3:**
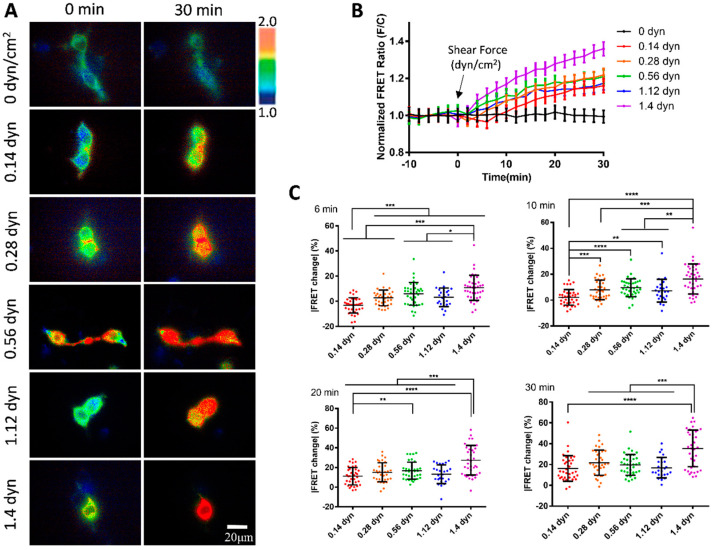
**vWF-based FRET responses under micro-shear forces from 0.14 to 1.4 dyn/cm^2^.** (**A**) Representative ratiometric FRET images of 293T cells expressing vWF-based biosensor at 0 and 30 min after applying 0, 0.14, 0.28, 0.56, 1.12, and 1.4 dyn/cm^2^ micro-shear flow. (**B**) Time-course curves of normalized FRET changes (mean ± S.E.M.) before and after the micro-shear flow (0.14–1.4 dyn/cm^2^), with 0 dyn/cm^2^ as control. (**C**) Statistical comparisons of FRET changes in percentages (mean ± S.E.M.) after applications of different micro-shear flows (0.14–1.4 dyn/cm^2^) at 6, 10, 20, and 30 min, respectively. Percentage values (mean ± S.E.M) at 30 min were as follows: 0.14 dyn, 16.16 ± 2.06, N = 36; 0.28 dyn, 21.71 ± 2.04, N = 35; 0.56 dyn, 19.68 ± 1.65, N = 37; 1.12 dyn, 16.90 ± 1.95, N = 25; 1.4 dyn, 35.48 ± 2.86, N = 38. N refers to sample size. Experiment had three independent repeats. *, **, ***, and **** represent *p*-values < 0.05, 0.01, 0.001, and 0.0001 for significant differences.

We further examined the mechanosensitivity of vWF under the scale of micro-shear forces. A gradual increment of micro-shear forces ranging from 0.14 to 1.4 dyn/cm^2^ was chosen to study the sensitivity of vWF-based FRET responses. The results showed that these micro-shear forces were sufficient to induce FRET responses from the conformation changes in the vWF-based biosensor (Figure 3A). Specifically, the FRET changes were positively correlated with the magnitudes of the micro-shear forces (0.14–1.4 dyn/cm^2^) (Figure 3B), demonstrating the ability of the cell membrane-anchored vWF-based biosensor in sensing the tiny hydrodynamic forces. By quantitatively comparing the responses to the gradient micro-shear forces, the FRET changes showed differences as early as 6 min after the flow applications with the lowest response from 0.14 dyn/cm^2^ (i.e., 1.4 μN/cm^2^), while 1.4 dyn/cm^2^ had a relatively higher response than the others through the 30 min flow processes (Figure 3C). These data indicate that the membrane-anchored vWF molecules are highly sensitive to the hydrodynamic forces.

### 3.4. Shear Force-Induced vWF FRET Response from A2 Conformation Change

To further investigate the molecular mechanism of shear force-induced vWF FRET response, we constructed an A2-LOCK version of the vWF biosensor in which the N- and C-terminals of the A2 domain were linked by a disulfide bond (Figure 1B). Our recent work also demonstrated the advantage of FRET application in verifying intermolecular interaction sites [52]. The previous study showed that the vWF mutant with LOCK-A2 exhibited attenuated vWF self-associations, collagen and platelet bindings, and ADMAMTS13 cleavage under shear flow [47]. To check whether the flow-induced FRET change was vWF-dependent, we included an MT1-MMP biosensor as a control which was also located in the pDisplay vector and presented on the cell surface as a non-plastic and small-sized protein [51].

**Figure 4:**
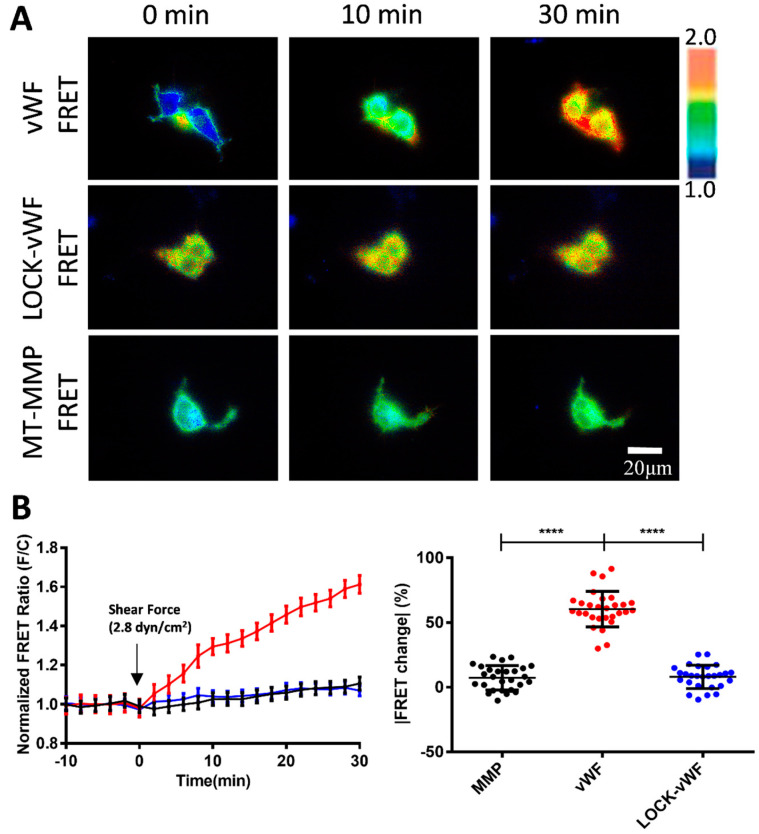
Comparisons for flow-induced FRET responses of vWF and LOCK-vWF-based biosensors as well as small-sized MT1-MMP biosensor. (**A**) Representative ratiometric FRET images of 293T cells expressing vWF or LOCK-vWF-bazsed biosensor, or MT1-MMP biosensor in response to 2.8 dyn/cm^2^ shear force. (**B**) Time-course curves (**left**) and scatter plots (at 30 min, **right**) of quantified FRET changes for cell groups in (**A**). Values of scatter plots (mean ± S.E.M.) are listed here: vWF-FRET, 60.49 ± 2.56, N = 29; LOCK-vWF-FRET, 7.36 ± 1.81, N = 27; MT1-MMP FRET, 8.08 ± 1.73, N = 27. N refers to sample size. **** represents *p*-value < 0.0001 for significant difference.

The flow at 2.8 dyn/cm^2^ induced about a 60% FRET change in the vWF-based biosensor in 30 min, but small FRET changes for the LOCK-vWF or MT1-MMP biosensors (Figure 4A,B, Movie S2). This indicates that the hydrodynamic force-induced FRET response depended on the specific feature of vWF molecules. The LOCK-A2 domain in the vWF biosensor could resist the flow-induced conformational change, suggesting that the A2 unfolding was important for the vWF sensing of the hydrodynamic force. The LOCK-vWF biosensor showed a higher basal FRET level (+27%) than the wild-type vWF one (Figure S1A), indicating that the disulfide bond-connected N- and C-terminals of the A2 domain had closer positioning than the wild-type one, or partially resulted in the more parallel positioning of the ECFP and YPet. The MT1-MMP biosensor showed little FRET response, which confirmed that the FRET change in vWF was not due to shear force on the FRET pair, and also suggested taht the large molecular size or plastic feature of vWF is crucial in the flow mechanosensation.

### 3.5. Response of A2-Only-Based FRET Biosensor to the Hydrodynamic Force

**Figure 5:**
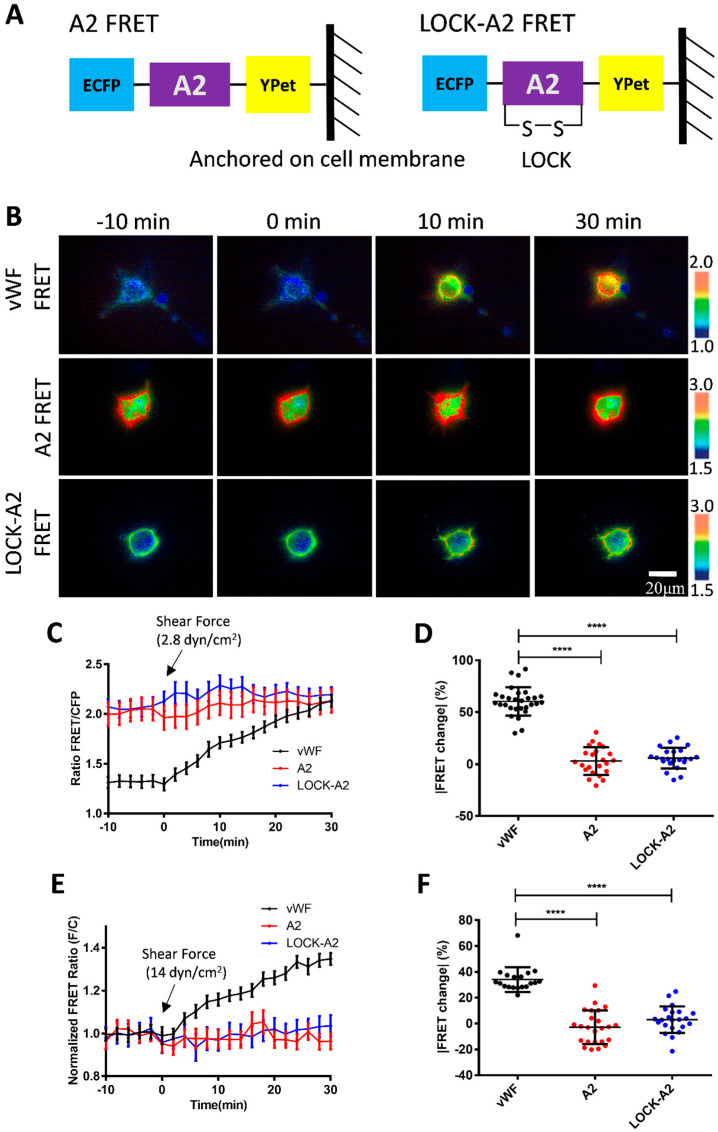
**The response of A2-based FRET biosensor to shear force.** (**A**) The design of FRET biosensors was based on A2-only and LOCK-A2, and both were anchored onto the surface of the cell plasma membrane. (**B**) Representative ratiometric FRET images of 293T cells expressing vWF FRET, A2 FRET, or LOCK-A2 FRET biosensors in response to 2.8 dyn/cm^2^ of shear force. (**C**,**D**) The time-course curves (**C**) and scatter plots (at 30 min, **D**) of FRET changes for the cell groups under the 2.8 dyn/cm^2^ condition (**B**). The percentage values of scatter plots (mean ± S.E.M) are as follows: vWF, 60.49 ± 2.55, N = 29; A2 only, 3.13 ± 2.76, N = 23; LOCK-A2 only, 5.92 ± 2.09, N = 23. (**E**,**F**) The time-course curves (**E**) and scatter plots (at 30 min, **F**) of FRET change quantifications for the indicated cell groups in response to 14 dyn/cm^2^. The percentage values of scatter plots in (**F**) (mean ± S.E.M): vWF, 34.14 ± 2.20, N = 19; A2 only, −2.77 ± 2.66, N = 24; LOCK-A2 only, 3.01 ± 2.19, N = 22. N refers to the sample size. The experiments were repeated three times, independently. **** represents *p*-value < 0.0001 for significant difference.

The A2 domain is the mechanosensitive element in vWF responding to the hydrodynamic force. Hence, we constructed an A2-based FRET biosensor to investigate whether the A2 domain only was able to sense the low-magnitude flow, or whether it depended on the large size of the vWF molecules. The expressed A2 biosensor and LOCK-A2 biosensor were anchored on the surface of cell plasma membrane (Figure 5A). Under a 2.8 dyn/cm^2^ shear force on the seeded cells in the fluidic channel, the vWF-based biosensor showed an apparent FRET response with an ~60% change in 30 min, whereas the A2 FRET or LOCK-A2 FRET biosensors did not have obvious FRET responses (Figure 5B–D, Supplementary Materials, Movie S3). It is noticed that the A2-only biosensor had a much higher FRET basal level (FRET/ECFP ratio: ~2.0, ~50% more) than that of the vWF-based biosensor (ratio: ~1.3) (Figure 5C and Figure S1B), which suggests a closer positioning of the N- and C-terminals in the A2-only one, or that vWF had a pulling force on the A2 domain in the full-length molecules. We further applied a higher shear force (14 dyn/cm^2^) onto the three versions of biosensors, which did not activate A2-only-based biosensor either (Figure 5E,F). Hence, at the low-scale flow rate (below 14 dyn/cm^2^), the plastic vWF with a large molecular size seems to be essential to sense the shear force and induce A2 unfolding.

## 4. Discussion

vWF has been recognized as a biomechanical probe that assists in the hydrodynamic force-induced coagulation and thrombus formation at the wound of vascular injury. Recent work provided the probable insight that the initial vWF-binding collagen at the injured subendothelial layer may be independent from the flow force, while the following binding of platelets relies on the shear-induced unfolding of vWF [53]. This hypothesis may help explain that free vWF is not sensitive to the regular flow in the circulation system, but anchored vWF at the injured sites becomes unfolded by shear force to further recruit platelets in assembling the clots. The A2 domain is established as a mechanosensitive element in vWF to promote conformation change and the elongation of this molecule in response to shear force [41,47]. In this work, we constructed a FRET-based biosensor anchored on the cell surface to monitor the vWF mechano-response to the hydrodynamic force, in which the A2 domain is flanked by an ECFP and YPet pair (Figure 1A–C). As a technology, FRET efficiency is highly sensitive to the spatial change between the pair, as inversely proportional to the sixth power of the distance [54], which can provide an untraditional way to monitor the shear-induced conformation change in vWF molecules.

We applied microfluidic channels to study the shear response of the vWF-based FRET biosensor presenting on the cell surface. Fluid shear is closely related to the developmental processes and physiological activities, and has important impacts on a variety of cardiovascular and hematologic diseases. How shear stress is involved in regulating the function of vWF proteins has been a hot topic in research. The results from our FRET measurements showed significant response of the biosensor to the hydrodynamic force, with a 60% FRET change at 2.8 dyn/cm^2^ in 30 min, which is higher than the 38% change at 1.4 dyn/cm^2^, but the FRET response did not continue to increase under further enhanced flow from 7 to 28 dyn/cm^2^, but, in contrast, started to decrease (Figure 2A–C). When we applied a gradient micro-shear flow below 1.4 dyn/cm^2^, the FRET response was still obvious at 0.14 dyn/cm^2^, and displayed certain gradual changes from 0.14 to 1.4 dyn/cm^2^ (Figure 3A–C). These FRET measurements indicate the high sensitivity of vWF in responding to shear force even under 1.4 dyn/cm^2^, which seems consistent with the clot formation at minor skin wounds. In comparison to some previous visualizations of vWF elongation under a higher shear rate (tens to hundreds of dyn/cm^2^), this FRET-based study of vWF unfolding was able to sensitively monitor the vWF conformation change under a low shear force (several dyn/cm^2^).

As mentioned in the Results, the flow induced an increase in FRET efficiency between ECFP and YPet (Figure 2 and Figure 3), indicating a closer positioning of N- and C-terminals of the A2 domain, or the addition of a more parallel orientation of ECFP and YPet. This measured result seems contradictive to the predicted unfolding of the A2 domain in vWF by high shear force. Hypothetically, from a different angle, considering that the A2 domain binds to the A1 domain or adds A3 as an intramolecular autoinhibitory mechanism for vWF [39,55,56], the flow induces dissociation of A2 from A1 binding during vWF unfolding, which results in the closer positioning of the N- and C-terminals of the A2 domain. In supporting this, the A2-only biosensor showed a much higher FRET level (50% more in ratio) than that of the vWF-based biosensor (Figure 5C), indicating a closer positioning of the two terminals of the released A2 domain without A1 or A3 binding.

It is interesting to notice that the micro-flow induced a gradual increase in the FRET efficiency at a low shear scale of 0–2.8 dyn/cm^2^, which then started to decline from 2.8 to 28 dyn/cm^2^ (Figure 2C). The observations from FRET measurements seemed to point to the two-level unfolding of vWF: the A2 dissociation from vWF intramolecular binding of A1/A3 domains at a low shear force, and then the unfolding of the A2 domain under a higher shear force for cleavage accessed by ADAMTS13. This conclusion drawn from FRET measurement may not be a surprise from studying previous studies. First, the mutual interaction of A domains in vWF has been demonstrated experimentally [57], and the application of a stretching force of ~20 pN by an optical trap elongated the engineered protein fragment consisting of three repeats of (A1-A2-A3) domains [58]. Second, single-molecular experiments with laser tweezers proved the mechanoenzymatic cleavage of A2 in vWF by ADAMTS13, in which only the stretch-unfolded A2 domain is accessible to the enzymatic cleavage [59]; the LOCK-A2 domain that prevents A2 unfolding reduced vWF self-association and binding of platelets under flow shear [47]. Third, single-molecular stretch did show two unfolding events or extension peaks of vWF, specifically at the pulling forces of ~23 pN and ~40 pN [58]. Although the previous work did not distinguish the intramolecular dissociation of A domains in vWF from A2 unfolding, our FRET measurements from the designed vWF biosensor could help reveal the two-level unfolding of vWF by the spatial intramolecular conformation changes.

Previous studies have shown that the cause of some vascular hemophilia is due to the inability of the A2 structural domain in vWF to undergo a conformational change in a patient as compared to a normal human. The A2 domain is the mechanosensitive element in vWF, and the LOCK-A2 domain (with a disulfide-bond bridging of its N- and C-terminals) could resist the shear flow-induced vWF self-association and ADAMTS13 cleavage [47]. We generated a version of a vWF-based FRET biosensor containing LOCK-A2 (LOCK-vWF FRET), and also introduced an MT1-MMP biosensor as a control which is located on the cell surface but with a small molecular size [51]. Under 2.8 dyn/cm^2^ of flow, vWF FRET showed a 60% FRET change in 30 min, while the LOCK-vWF (with a higher basal FRET ratio) or MT1-MMP biosensors showed very little FRET change (Figure 4A,B). These results demonstrated that the structure-flexible A2 domain is essential for the flow-induced conformation change, and confirmed that the observed FRET change in the vWF biosensor was not from shear force exerted on the ECFP/YPet pair.

We further constructed an A2-only FRET biosensor and its LOCK-A2 version, which did not show obvious FRET responses to 2.8 and 14 dyn/cm^2^ (Figure 5A–F). An earlier study reported that chemical denaturation of the A2 domain by 6 M of Urea caused the unfolding of the A2 domain and a decrease in the FRET efficiency of an A2-based biosensor [60]. Our data indicates that A2 alone is not sensitive to the hydrodynamic force (14 dyn/cm^2^), and the integrity of full-length vWF reserves the ability of a high mechano-sensitivity to shear force even at a scale as low as 0.14 dyn/cm^2^. It is noted that the A2-only biosensor had a much higher FRET level (ratio: ~2.0) than that of the vWF-based biosensor (ratio: ~1.3) (Figure 5C). This seems consistent with the shear-induced FRET increase in the vWF biosensor, which may be resulted from the shear-induced dissociation of the A2 domain from the binding of A1 and A3 domains.

To compare the FRET changes under varying shear forces to some previously published results of their measurements, we found certain consistency. One recent report showed that glass-tethered vWF molecules had subtle extensions under 20 dyn/cm^2^ and started to rapidly increase their length above 20 dyn/cm^2^ [54], while our FRET measurements of the cell-surface-anchored vWF-based biosensor indicated that there was little A2 unfolding below 2.8 dyn/cm^2^ and more A2 unfolding around 14–28 dyn/cm^2^ at a similar shear scale (Figure 2). From calibrations, 28 dyn/cm^2^ of the shear stress in our system corresponded to 2641/s in shear rate. For comparison, one previous report showed that fluorescent vWF molecules attached onto the glass started dramatic extensions around the shear rate of 2000/s, but less obvious below 1000/s [34]. Another report demonstrated similarly that wild-type vWF demonstrated a big difference in recruiting platelets compared to the A2-Locked vWF at shear rate of 2000/s, but they were less different below 1000/s, likely resulting from A2 unfolding [48]. Our FRET data proved a certain consistency with these two reports, which showed obvious A2 unfolding around a shear rate of 2000/s (14–28 dyn/cm^2^ in Figure 2). In reported single molecular studies with optical tweezers or atomic force microscopy, the two ends of vWF molecules were fixed, which generally identified a single A2 unfolding within 10–30 pN of stretch [35,58,59]. It is noted that the experimental setups were somewaht different from the flow shear condition with one free vWF end.

FRET efficiency can be influenced by the relative orientation of the FRET donor and acceptor despite their distance. It is possible that the shear flow-induced FRET change could be partially contributed from the orientation shift between ECFP and YPet. In considering that wild-type vWF and LOCK-vWF biosensors shared similar structures, the LOCK-vWF biosensor as negative a control had almost no FRET change while the vWF-based one had an ~60% increase under 2.8 dyn/cm^2^ of shear flow, hence the increase from the later one was more likely contributed from the distance change between the FRET pair, instead of their orientational change. The flow-induced conformational changes in the vWF-based biosensor need be further verified by other alternative tools, such as computer simulation, analysis by small-angle X-ray scattering, or cryo-electronic microscopy.

Regarding one concerning point that the vWF molecule may be modified from its natural structure in the biosensor, can provide some additional clarifications: (1) To avoid the structural interference, the ECFP and YPet fluorescent proteins are added into the flexible regions between the A1, A2, and A3 domains in vWF, and both ECFP and YPet proteins have their own flexible peptides at their N- and C-terminals. (2) The monitored vWF two-level unfolding in the biosensor by shear fluid was similarly observed in the previously published results performed with intact vWF single molecules by optical tweezers, which displayed two-stage extensions of the vWF [59]. Hence, we think that, probably, the vWF in the biosensor has basically conserved its structural and functional integrity under the current study condition.

## 5. Conclusions

**Figure 6:**
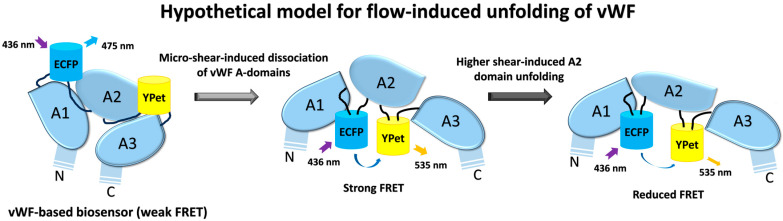
**Hypothetical model of shear-induced vWF unfolding.** Based on the data from this work along with previous studies, shear flow induces two-level unfolding of vWF-based biosensor in hypothesis: the dissociation of three A domains in vWF molecules under low shear force, which results in an increase in FRET efficiency in the vWF-based biosensor; followed by the unfolding of A2 domain under higher shear force, which results in declining FRET efficiency.

In summary, we developed a shear force biosensor based on the mechanosensitive nature of vWF molecules. By combining FRET imaging with a microfluidic channel, the biosensor anchored onto the cell surface displayed high sensitivity to the hydrodynamic force with increasing FRET responses along with micro-shears from 0.14 to 2.8 dyn/cm^2^, which started to decline at a higher magnitude range of 2.8–28 dyn/cm^2^. The gradient shear-induced increase and then decrease in FRET efficiency indicates the two-level unfolding of vWF: the dissociation of the A2 domain from vWF intramolecular binding under a low shear, which is supported with the stronger FRET of the A2-only-based biosensor, and then the unfolding of the A2 domain in vWF under a higher shear, which is consistent with previous work (illustrated Figure 6). The LOCK-vWF biosensor lost its FRET response to the hydrodynamic force, indicating that a structure-flexible A2 if important for force mechanosensation. The FRET biosensor containing A2 only could not respond to the shear force under 14 dyn/cm^2^, implying that the large vWF molecule is needed for the normal physiological functions during shear-induced coagulation and thrombus assembly. The vWF-based biosensor demonstrated the high mechanosensation of vWF to shear force even at a low scale (0.14–1.4 dyn/cm^2^), which may support the observed clot formation at microvascular wounds. In addition to the previous biomechanical studies of vWF, the FRET technology shows high sensitivity and experimental convenience in monitoring the conformation change in vWF molecules.

Additionally, the FRET images showed the vWF biosensor on the cell surface experiencing the most shear forces, while there was also a FRET change inside the cells, either from the biosensor derived from the surface or induced by shear force within cells. The dense vWF biosensors in cells might have an impact on the FRET response from inter-molecular FRET, or the change in the force-sensing nature due to vWF inter-molecular interaction, and multimer formation. Further research is also needed to clarify the underlying molecular mechanisms for the shear-induced two-level unfolding of vWF. In vitro study by purifying the vWF-based biosensor protein can help validate the vWF’s function in binding to collagen and platelets. FRET imaging can also be moved to vascular cells as they are more physiologically relevant, or those with a higher flow rate. As an additional factor, further input of the ADAMTS13 enzyme in the flow may help us to understand its role in vWF mechanosensation and conformation change.

## Data Availability

Data are contained within the article and Supplementary Materials, or available upon reasonable request to the corresponding author.

## Supplementary Materials

The following supporting information can be downloaded at: https://www.mdpi.com/article/10.3390/bios15040248/s1, Figure S1: Comparisons of basal FRET levels. (A,B) Statistical comparisons of the basal FRET levels before shear flow for vWF-based biosensor with LOCK-vWF biosensor, MT1-MMP biosensor (A), and with A2 only biosensor, LOCK-A2 biosensors (B). Mean ± S.E.M. for vWF: 1.32 ± 0.059; LOCK-vWF: 1.68 ± 0.054; MT1-MMP: 1.20 ± 0.061; A2: 2.03 ± 0.11; LOCK-A2: 2.06 ± 0.084. Movie S1. FRET changes of vWF-based biosensor in 293T cells under fluid shear of 2.8 dyn/cm^2^, with 0 dyn/cm^2^ as control. After 10 min of FRET imaging before flow, the peristaltic pump was turned on for 30 min of flow administration. Fluorescence images were acquired at 2-min interval. Movie S2. Comparison for flow-induced FRET responses of vWF and LOCK-vWF-based biosensors in responding to 2.8 dyn/cm^2^ shear force. Time interval = 2 min. Movie S3. The response of A2 only-based FRET biosensor to shear force. vWF FRET, A2 FRET, or LOCK-A2 FRET biosensor was anchored on the surface of 293T cell plasma membrane in responding to 2.8 dyn/cm^2^ shear force. Time interval = 2 min.
