# Supplementary material for: FRET Visualization of High Mechanosensation of von Willebrand Factor to Hydrodynamic Force
Source: Biosensors (Basel). 2025 Apr 14;15(4):248. doi: 10.3390/bios15040248 (PMC12026062; doi:10.3390/bios15040248)
Supplement: Supplementary file 1 [file biosensors-15-00248-s001.zip › biosensors-3492871-supplementary/biosensors-3492871-supplementary/Supplementary information/Supporting Information text.pdf]

Supporting Information text

FRET visualization of high mechanosensation of von Willebrand factor to hydrodynamic force

Mingxing Ouyang[1,#]*, Yao Gao[1.2#], Binqian Zhou[1,2], Jia Guo[1], Lei Lei[3], Yingxiao Wang[3,4], Linhong Deng[1,]*

[1] Institute of Biomedical Engineering and Health Sciences, School of Medical and Health Engineering, Changzhou University, Changzhou, Jiangsu Province 213164 China

[2] School of Pharmacy, Changzhou University, Changzhou, Jiangsu Province 213164 China

[3] Shu Chien-Gene Lay Department of Bioengineering, and Institute of Engineering in Medicine, University of California at San Diego, La Jolla, CA 92093 USA

[4] Alfred E. Mann Department of Biomedical Engineering, University of Southern California, Los Angeles, CA 90089 USA

Supporting information contains one figure and three movies.

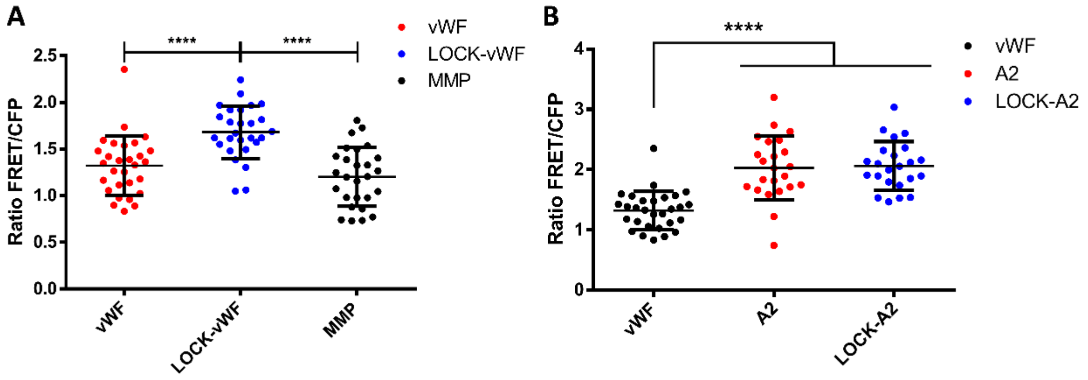

Figure S1. Comparisons of basal FRET levels. (A, B) Statistical comparisons of the basal FRET levels before shear flow for vWF-based biosensor with LOCK-vWF biosensor, MT1-MMP biosensor (A), and with A2 only biosensor, LOCK-A2 biosensors (B). Mean±S.E.M. for vWF: 1.32±0.059; LOCK-vWF: 1.68±0.054; MT1-MMP: 1.20±0.061; A2: 2.03±0.11; LOCK-A2: 2.06±0.084.

Supplementary movie legends

Movie S1. FRET changes of vWF-based biosensor in 293T cells under fluid shear of 2.8 dyn/cm^2, with 0 dyn/cm^2 as control. After 10 minutes of FRET imaging before flow, the peristaltic pump was turned on for 30 minutes of flow administration. Fluorescence images were acquired at 2-minute interval.

Movie S2. Comparison for flow-induced FRET responses of vWF and LOCK-vWF-based biosensors in responding to 2.8 dyn/cm^2 shear force. Time interval = 2 min.

Movie S3. The response of A2 only-based FRET biosensor to shear force. vWF FRET, A2 FRET, or LOCK-A2 FRET biosensor was anchored on the surface of 293T cell plasma membrane in responding to 2.8 dyn/cm^2 shear force. Time interval = 2 min.
